# 40 The effects of acute exercise on emotional pattern separation in adolescents and young adults

**DOI:** 10.1093/eurpub/ckae114.113

**Published:** 2024-09-26

**Authors:** Lise Jennen, Victor Mazereel, Davy Vancampfort, Zhiling Qiao, Kristof Vansteelandt, Patrick Dupont, Aleksandra Lecei, Ruud van Winkel

**Affiliations:** KU Leuven, Department of Neurosciences, Center for Clinical Psychiatry, Leuven, Belgium; KU Leuven, Department of Neurosciences, Center for Clinical Psychiatry, Leuven, Belgium; University Psychiatric Center KU Leuven, Leuven, Belgium; University Psychiatric Center KU Leuven, Leuven, Belgium; KU Leuven Department of Rehabilitation Sciences, Leuven, Belgium; KU Leuven, Department of Neurosciences, Center for Clinical Psychiatry, Leuven, Belgium; University Psychiatric Center KU Leuven, Leuven, Belgium; KU Leuven, Department of Neurosciences, Laboratory for Cognitive Neurology, Leuven, Belgium; KU Leuven, Department of Neurosciences, Center for Clinical Psychiatry, Leuven, Belgium; KU Leuven, Department of Neurosciences, Center for Clinical Psychiatry, Leuven, Belgium; University Psychiatric Center KU Leuven, Leuven, Belgium

## Abstract

Exercise has been widely recognized for its benefits on cognitive health. One potential underlying mechanism is the modulation of hippocampal pattern separation (PS), a memory process crucial for accurate storage and preventing memory interference. PS can be gauged behaviorally with a mnemonic discrimination task using images, and neurally with functional magnetic resonance imaging assessing hippocampal activation of primarily the dentate gyrus and CA3 subregions.

While a number of studies have demonstrated a positive effect of acute exercise on PS, the majority of studies only investigated PS behaviorally. Furthermore, these previous studies examined PS using neutral images, but it is often the recollection of emotional experiences that guides behavior. Additionally, research in this area predominantly focused on adult populations, neglecting the critical developmental period of adolescence and young adulthood, characterized by decreased exercise and increased vulnerability to emotional problems.

In this randomized between-subject study involving healthy adolescents and young adults (n = 53), we extend previous findings by examining the effect of a 10-minute bout of moderate-intensity exercise intervention compared to rest on both behavioral and neural PS, utilizing emotional images. Task-related neuronal activity was discerned from exercise induced changes in cerebral blood flow by including arterial spin labeling scans. We hypothesized increased behavioral mnemonic discrimination ability and neural hippocampal PS activation after the exercise intervention compared to rest. Across interventions, we hypothesized decreased discrimination for emotional images and more similar images.

Contrary to our hypotheses, we could not demonstrate a significant benefit of acute exercise on PS, not on the behavioral or neural level. Behaviorally, we did observe decreased mnemonic discrimination for more similar images and for highly-similar positive images. However, correlation analysis did not demonstrate an association between behavioral discrimination ability and neural activity, and exploratory analysis did not reveal a neural signal consistent with pattern separation in any of the hippocampal subregions.

In conclusion, this study underscores the need to broaden our hippocampal focus of PS, in order to bridge behavioral mnemonic discrimination with neural PS activation. In addition, future studies should optimize the exercise characteristics necessary to robustly enhance PS.

* LJ and VM shared first authorship

